# Effects of Mono- and Bifunctional Surface Ligands of Cu–In–Se Quantum Dots on Photoelectrochemical Hydrogen Production

**DOI:** 10.3390/ma15176010

**Published:** 2022-08-31

**Authors:** Soo Ik Park, Sung-Mok Jung, Jae-Yup Kim, Jiwoong Yang

**Affiliations:** 1 Department of Energy Science and Engineering, Daegu Gyeongbuk Institute of Science and Technology (DGIST), Daegu 42988, Korea; 2 Department of Chemical Engineering, Dankook University, Yongin 16890, Korea; 3 Energy Science and Engineering Research Center, Daegu Gyeongbuk Institute of Science and Technology (DGIST), Daegu 42988, Korea

**Keywords:** photoelectrochemical, hydrogen generation, CuInSe_2_, quantum dots, surface ligands, surface engineering, photoanodes

## Abstract

Semiconductor nanocrystal quantum dots (QDs) are promising materials for solar energy conversion because of their bandgap tunability, high absorption coefficient, and improved hot-carrier generation. CuInSe_2_ (CISe)-based QDs have attracted attention because of their low toxicity and wide light-absorption range, spanning visible to near-infrared light. In this work, we study the effects of the surface ligands of colloidal CISe QDs on the photoelectrochemical characteristics of QD-photoanodes. Colloidal CISe QDs with mono- and bifunctional surface ligands are prepared and used in the fabrication of type-II heterojunction photoanodes by adsorbing QDs on mesoporous TiO_2_. QDs with monofunctional ligands are directly attached on TiO_2_ through partial ligand detachment, which is beneficial for electron transfer between QDs and TiO_2_. In contrast, bifunctional ligands bridge QDs and TiO_2_, increasing the amount of QD adsorption. Finally, photoanodes fabricated with oleylamine-passivated QDs show a current density of ~8.2 mA/cm^2^, while those fabricated with mercaptopropionic-acid-passivated QDs demonstrate a current density of ~6.7 mA/cm^2^ (at 0.6 V_RHE_ under one sun illumination). Our study provides important information for the preparation of QD photoelectrodes for efficient photoelectrochemical hydrogen generation.

## 1. Introduction

Solar energy is a promising sustainable energy resource owing to its infinite supply and low environmental impact. Specifically, the Sun continuously delivers an enormous energy of 1.7 × 10^5^ TW to Earth, which is several orders of magnitude larger than that produced by human civilization. It is highly desirable to develop efficient methods to convert photons into electricity, chemicals, and heat, and this has inspired tremendous interest in research on solar cells [1,2], photocatalysts [3,4,5,6], and photoelectrochemical (PEC) devices [7,8,9,10,11,12,13]. Among these various techniques, PEC hydrogen production provides sustainable and cost-effective methods for direct solar-to-chemical energy conversion to produce clean solar fuels. Previous studies on PEC hydrogen production typically used metal oxide materials such as TiO_2_, BiVO_4_, Fe_2_O_3_, and WO_3_ because of their low cost and high stability during water splitting. However, their wide bandgaps (e.g., 3.2 eV for TiO_2_:) inhibit the effective utilization of the full solar spectrum.

Additional light absorbers with narrower bandgaps have been introduced to solve the problems of wide-bandgap oxide semiconductors [14,15,16,17,18,19,20,21,22]. These absorbers can use the light that cannot be absorbed by wide-bandgap oxide materials to generate more photoexcited electrons, which are then transferred to the oxide materials for further photocatalytic reactions. Semiconductor nanocrystal quantum dots (QDs) have been regarded as promising absorbers because of their unique properties such as size- and shape-dependent bandgap tunability [23,24,25,26], high absorption coefficient [27], and multiple exciton carrier generations [28,29]. Among these, heavy-metal-free I–III–VI QDs, such as CuInSe_2_ (CISe) QDs, are environmentally benign and can effectively absorb visible and near-infrared spectral regions, making them one of the ideal candidates for solar-to-chemical conversion [30,31,32,33,34,35,36]. Studies on PEC hydrogen production using these QDs are recent [37,38,39], implying that extensive research is necessary before they can be used in practical applications.

Colloidal QDs are generally synthesized in colloidal solutions [40] and are composed of inorganic crystalline nanoparticles and organic surfactants that passivate the surface of the nanoparticles. These surface ligands have multiple functions, including controlling the synthesis process, stabilizing the QDs, regulating the solution dispersibility, and controlling the optical and electrical properties of the QDs [41,42,43]. Because of their significant impact on the properties of QDs, surface ligands are carefully controlled to fully exploit the unique properties of QDs. Surface ligands should be selected by considering the role of the QDs and the fabrication process for the target applications. For example, to enhance charge transport between QDs, the use of short-chain ligands is generally preferred for QD solar cells [44,45]. However, despite their importance, the effect of the surface ligands of QDs on their PEC applications has been less studied.

In this study, we investigated the effects of the surface ligands of colloidal CISe QDs on the fabrication of PEC photoanodes and the resulting PEC characteristics. CISe QDs passivated with monofunctional oleylamine (OAm) were synthesized by colloidal synthesis. Through a post-ligand exchange process, QDs passivated with mercaptopropionic acid (MPA, bifunctional surface ligands) were prepared for comparison. Photoanodes for PEC hydrogen production were prepared by adsorbing QDs on a mesoporous TiO_2_ film, which had two different QD adsorption mechanisms according to the choice of surface ligands. Monofunctional ligand-passivated QDs were directly attached on TiO_2_ by partial ligand detachment, enhancing electron transport between the QDs and TiO_2_. Bifunctional ligands acted as linkers by bridging QDs and TiO_2_, and the amount of QD adsorption was higher for MPA-passivated QDs than for OAm-passivated ones. With this trade-off, photoanodes fabricated with OAm-passivated QDs and those with MPA-passivated QDs demonstrated photocurrent densities of ~8.2 and ~6.7 mA/cm^2^, respectively (at 0.6 V_RHE_, one sun illumination). We believe that our results will contribute to the development of systems with effective PEC hydrogen generation using colloidal QDs.

## 2. Materials and Methods

### 2.1. Materials

Copper(I) iodide (CuI, 99.998%), indium(III) iodide (InI_3_, 99.999%), and 3-mercaptopropionic acid (MPA, 99%) were purchased from Alfa-Aesar. Dichloromethane (99.8%), oleylamine (OAm, technical grade), oleic acid (OAc, technical grade), trioctylphosphine (TOP, 97%), selenium (99.99%), 1-dodecanethiol (DDT, 98%), 1-octylamine (OcAm, 99%), zinc nitrate hexahydrate (Zn(NO_3_)_2_·6H_2_O, 98%), sodium sulfide (Na_2_S), and sodium sulfite (Na_2_SO_3_, ≥98.0%) were purchased from Sigma-Aldrich. Ethanol (99.5% anhydrous), methanol (99.5%), chloroform (99.95%), and sodium hydroxide (NaOH, 99.8%) were purchased from Samchun Chemicals. n-Butanol (99%) was purchased from Daejung Chemicals & Metals. The OAm was dried under a vacuum before use.

### 2.2. Synthesis of CISe QDs

Uniform-sized CISe QDs were synthesized according to our previously reported method [31,33]. In a typical synthesis, a metal-OAm complex precursor solution was prepared by heating 0.5 mmol of CuI and 0.5 mmol of InI_3_ in 15.0 mL of OAm at 120 °C under a vacuum for 30 min. An oleylammonium selenocarbamate precursor solution was prepared by heating 5.0 mmol of Se in OAm (10.0 mL) under a CO atmosphere at 80 °C. The metal–OAm complex solution was cooled to 70 °C, and 2.0 mL of the oleylammonium selenocarbamate solution was quickly injected into the solution with Ar flow. The reaction temperature was gradually increased to 180 °C and was maintained for 20 min. After the reaction, the QDs were precipitated via centrifugation using ethanol containing TOP to remove the remaining Se precursors. Finally, the QDs were dispersed in 4.0 mL of dichloromethane for further use.

### 2.3. Ligand Exchange Treatment of CISe QDs

For the ligand exchange of QDs from OAm to MPA, the phase-transfer ligand exchange process was used [46]. In a typical process, 1.3 mL of MPA was mixed with 4.0 mL of methanol and 40 wt% NaOH aqueous solution of the controlled amount. The total volume of the mixture was adjusted to 8.0 mL. The pH of the solution was controlled by adjusting the amount of aqueous NaOH solution. The QD solution was mixed with the MPA solution and stirred for 10 min. To remove the detached OAm, the mixture was washed several times with chloroform. Finally, the MPA-passivated QDs were dispersed in water.

For the ligand exchange of QDs from OAm to other monofunctional ligands such as OcAm, OA, and DDT, the single-phase ligand exchange process was used. In a typical process to prepare OcAm-passivated CISe QDs, 2.0 mL of OcAm was mixed with 1.0 mL of QD solution (40 mg/mL in dichloromethane). The mixture was vigorously stirred for ~2 h at 25 °C. The products were precipitated via centrifugation using ethanol and re-dispersed in dichloromethane for further use. Instead of OcAm, DDT and OAc were used, respectively, for the preparation of DDT- and OAc-passivated CISe QDs.

### 2.4. Fabrication of CISe QD-Sensitized TiO_2_ Photoanodes

Fluorine-doped tin oxide (FTO) glass (TEC-A7, Pilkington) was washed in ethanol under ultrasonication for 20 min, followed by treatment with UV/O_3_ (Yuil Ultraviolet System) for 15 min to remove any contaminants. Titanium diisopropoxide-bis(acetylacetonate) (7.5 wt%, Aldrich) in n-butanol was spin-coated on the surface of the washed FTO glass and subsequently annealed at 475 °C for 10 min in air. A nanocrystalline TiO_2_ paste (Ti-Nanoxide T/SP, Solaronix) was coated on the pretreated FTO glass using the doctor-blade method, followed by annealing at 525 °C for 30 min in air. Finally, the annealed FTO/mesoporous TiO_2_ film was immersed in a colloidal CISe QD solution (4.0 mg/mL) for 3 h for sensitization and then rinsed with dichloromethane. The ZnS overlayers were coated on the surface of the QD-sensitized TiO_2_ film by successive ionic layer adsorption and reaction (SILAR) processes, consisting of immersing the QD-sensitized TiO_2_ film in a 0.05 M Zn(NO_3_)_2_·6H_2_O ethanol solution and 0.05 M Na_2_S in a mixed solvent of deionized water/methanol (volume ratio = 1:1) for 1 min each. The SILAR process was repeated thrice.

### 2.5. Material Characterization

The absorption spectra of the CISe QDs were measured using a PerkinElmer Lambda 465 instrument. Time-resolved photoluminescence measurements were performed with a Horiba Fluoromax plus a time-correlated single-photon counting system using a DeltaDiode DD-375L laser diode (peak wavelength: 371 nm). The X-ray diffraction (XRD) patterns were acquired on a Horiba Miniflex 600 X-ray diffractometer. Transmission electron microscopy (TEM) images were obtained using an FEI Tecnai G2 F20 Twin TMP microscope. Fourier-transform infrared (FT-IR) spectroscopy analysis was performed using an Agilent Cary 660 FT-IR spectrometer in attenuated total reflectance measurement mode. X-ray photoelectron spectroscopy (XPS) was performed using a Thermo Scientific ESCALAB 250XI instrument.

### 2.6. Photoelectrochemical Measurements

All of the PEC measurements were carried out in a quartz reactor using a potentiostat (Multi Autolab M204, Metrohm) with a three-electrode system consisting of a QD-sensitized TiO_2_ film as the photoanode, a platinum mesh as the counter electrode, and a Hg/HgO (saturated calomel electrode, SCE) as the reference electrode. The electrolyte was composed of 0.25 M Na_2_S and 0.35 M Na_2_SO_3_ (pH ~12.9) in deionized water. Linear sweep voltammetry (LSV) measurements were performed at a scan rate of 20 mV/s under simulated light with one sun intensity (100 mW/cm^2^) using a solar simulator (PEC-L01, Peccell) with an AM 1.5G filter. Incident photon-to-current conversion efficiency (IPCE) spectra were obtained using a xenon lamp (300 W, Oriel), monochromator (TracQBasic 6.5, Oriel), and NIST-certified Si diode. Electrochemical impedance spectroscopy (EIS) measurements were performed using a frequency-response detector in the potentiostat under a sinusoidal perturbation of ± 10 mV in the frequency range of 0.1 Hz to 100 kHz.

## 3. Results and Discussion

### 3.1. Preparation of CISe QDs with Different Surface Ligands

**Figure 1:**
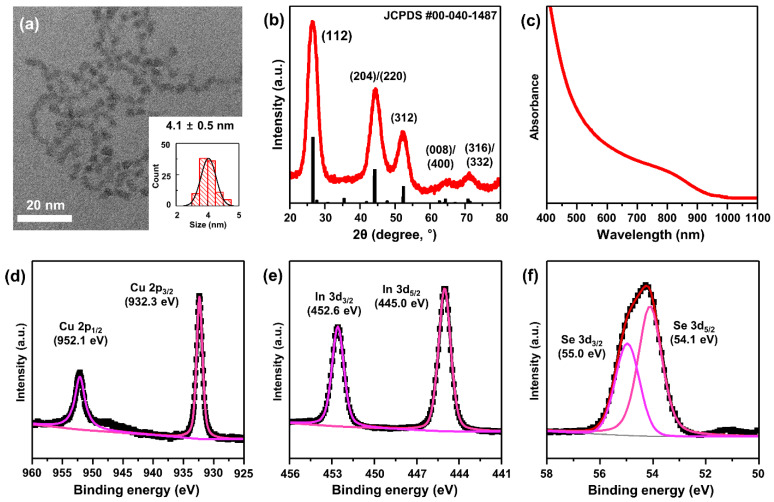
(**a**) TEM image of 4 nm-sized CISe QDs. Inset: histogram of the size distribution of the QDs (n = 100). (**b**) XRD pattern of CISe QDs. The reference XRD data of bulk chalcopyrite CuInSe_2_ crystals are also shown (JCPDS No.: 00-040-1487). (**c**) Absorption spectrum of CISe QDs. XPS spectra of (**d**) Cu, (**e**) In, and (**f**) Se from the CISe QDs.

To understand the effect of the surface ligand molecules of QDs on PEC hydrogen production, the CISe QDs were prepared by colloidal synthesis following our previous study [31,33] using the reaction between metal–ammine complexes and oleylammonium selenocarbamate (Materials and Method 2.2). As shown in the TEM image (Figure 1a), the synthesized QDs had an average size of ~4 nm with a narrow size distribution (standard deviation: 0.5 nm). From our previous work on solar cells using CISe QDs, ~4 nm was the optimum size for light absorption and electron transfer to TiO_2_, which was the main reason that 4-nm CISe QDs were used for this study. The XRD results confirm the tetragonal chalcopyrite crystal structure of the QDs (Figure 1b). The absorption spectrum (Figure 1c) and corresponding Tauc plot (Figure S1, Supplementary Materials) of the CISe QD solution show that the optical bandgap of the QDs was ~1.4 eV, which is appropriate for absorption of the full solar spectrum.

These QDs had a composition of CuIn_1.5_Se_3_, as revealed by inductively coupled plasma-atomic emission spectrometry (ICP-AES) analysis. It is known that smaller CISe QDs usually have higher In/Cu ratios because the surfaces of these nanocrystals preferably have In-rich states [30,31]. The electronic state of the CISe QDs was further investigated by XPS. The main peaks of the Cu 2p_3/2_ and Cu 2p_1/2_ XPS spectra of these QDs are located at 932.3 and 952.1 eV, respectively (Figure 1d), corresponding to the Cu^+^ oxidation state [47]. The estimated binding energies of In 3d_5/2_ and In 3d_3/2_ are 445.0 and 452.6 eV, respectively (Figure 1e), implying the In^3+^ oxidation state [48]. In addition, the XPS spectrum of Se shows that the main peaks of Se 3d_5/2_ and Se 3d_3/2_ were located at binding energies of 54.1 and 55.0 eV, respectively, which match well with those of Se^2-^ anions, while it does not have peaks corresponding to SeO_x_ that can be formed by surface oxidation. These data support the successful synthesis of high-quality chalcopyrite-structured CISe QDs without severe surface oxidation.

**Figure 2:**
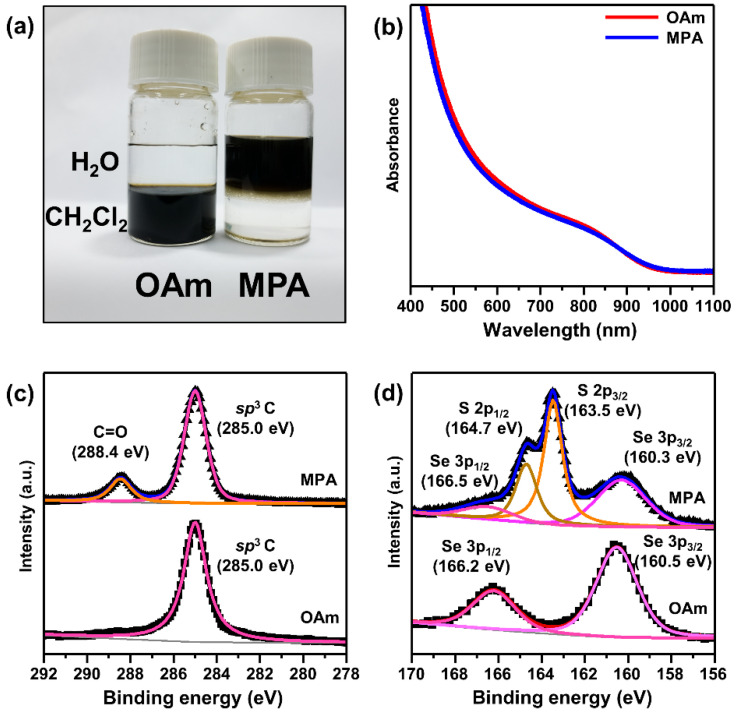
(**a**) Photograph of the two-phase ligand-exchange process. The bottom and top liquid layers are dichloromethane and water, respectively. OAm- and MPA-passivated QDs can be dispersed in dichloromethane and water, respectively. (**b**) Comparison of the absorption spectra of OAm- and MPA-passivated CISe QDs. XPS data for (**c**) C 1s and (**d**) S 2p, showing comparison between OAm- and MPA-passivated CISe QDs.

A post-ligand-exchange treatment was carried out to obtain CISe QDs with controlled surface ligand molecules. The as-synthesized CISe QDs were solely passivated by OAm because only OAm was used as a coordinating solvent for the synthesis without adding other organic surfactants. To obtain QDs passivated by bifunctional ligands, a two-phase ligand-exchange reaction was used to replace OAm with MPA, which contains thiol and carboxyl groups (Figure 2a). The thiol group can strongly bind to the surface of QDs, and the additional carboxyl group can make QDs dispersible in polar solvents [46,49,50,51]. As shown in Figure 2a, the ligand-exchanged QDs were dispersible in water, supporting the successful replacement of surface ligands. The absorption spectra of the OAm- and MPA-passivated QDs were almost identical in terms of their energetic positions and shapes (Figure 2b). Furthermore, the TEM images (Figure S2, Supplementary Materials) and XRD pattern (Figure S3, Supplementary Materials) of the CISe QDs after the ligand exchange are very similar to those of the as-synthesized QDs. All of the data verified that the ligand exchange process does not degrade the QDs.

The successful preparation of CISe QDs with controlled ligand molecules was further verified by XPS. The binding energy of the C 1s XPS main peak of OAm-passivated QDs is ~285 eV (Figure 2c), originating from the hydrocarbon chain [52]. In contrast, the C 1s XPS spectrum of the MPA-passivated QDs has an additional peak located at 288.4 eV, which is attributed to the presence of the carboxylic group in MPA. In addition, the S 2p_3/2_ XPS spectrum of the MPA-passivated QDs has a clear main peak corresponding to the thiol group, whereas that of the OAm-passivated QDs does not have a peak at the corresponding binding energy (Figure 2d) [53]. These results support the successful preparation of OAm- and MPA-passivated QDs for further studies. To gain a better understanding of the effects of surface ligands, we also prepared various monofunctional ligand-passivated QDs, including OcAm-, DDT-, and OAc-passivated QDs (Figure S4, Supplementary Materials).

### 3.2. Properties of TiO_2_–CISe QD Photoanodes

**Figure 3:**
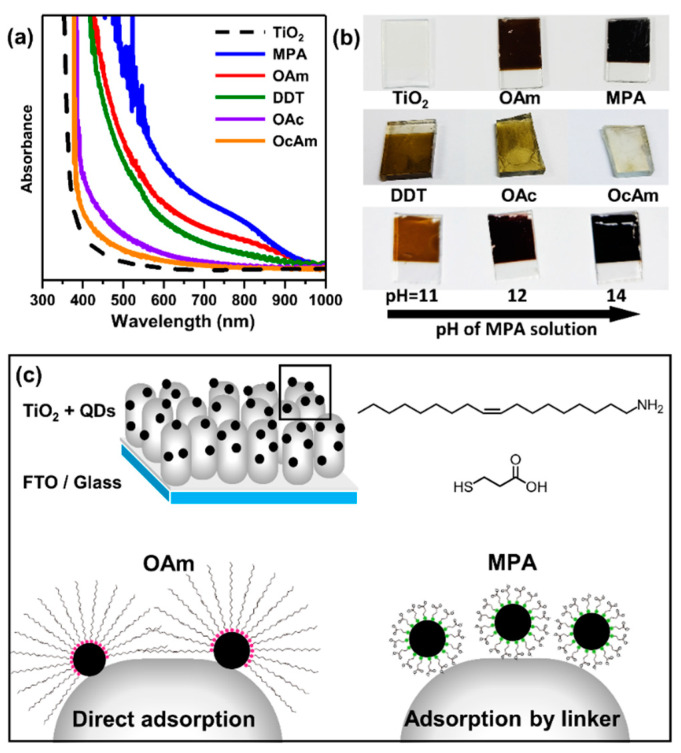
(**a**) Absorption spectra of bare and QD-sensitized TiO_2_ films using CISe QDs with various surface ligands. (**b**) Photographs of (top) bare, OAm-, MPA-, (middle) DDT-, OAc-, OcAm-passivated CISe QD-sensitized TiO_2_ films, and (bottom) CISe QD-sensitized TiO_2_ films made from MPA-passivated QDs as a function of the pH of the QD solution. (**c**) Schematic illustration showing the CISe QD-sensitized TiO_2_ film and the two different adsorption mechanisms of QDs.

To fabricate TiO_2_–CISe QD photoanodes, a mesoporous TiO_2_ film was dipped into a solution containing CISe QDs with controlled surface ligands (Materials and Methods 2.4). For both MPA- and OAm-passivated QDs, a dipping time of ~3 h in the QD solution resulted in the dense adsorption of QDs onto TiO_2_. A comparison between the absorption spectra of bare and CISe QD-sensitized TiO_2_ films suggests a significant enhancement of absorbance after dipping (Figure 3a), implying the successful adsorption of CISe QDs onto the TiO_2_ films. Cross-sectional scanning electron microscopy (SEM) images and elemental analysis results also support the sensitization of TiO_2_ by CISe QDs (Figures S5 and S6, Supplementary Materials). The absorbance in the ultraviolet range was higher after QD-sensitization, and the absorption wavelength was extended to the near-infrared range, suggesting the absorption of the full solar spectrum. Photographs of the TiO_2_–CISe QD photoanodes showed their deep brown and black colors, further supporting the strong adsorption of visible light by the QDs (Figure 3b).

We additionally tested various monofunctional ligand-passivated QDs, including OcAm-, OAc-, and DDT-passivated QDs (Figure S4, Supplementary Materials). However, for the reasons listed below, these QDs were not suitable for making PEC electrodes. The colloidal stability of the QDs was decreased by surface passivation with short-chain monofunctional ligands such as OcAm (C8) (Figure S4, Supplementary Materials). Furthermore, thiol or carboxylic groups adhere to the QD surface too strongly compared to the amine groups [54]. These factors prevented the effective sensitization of TiO_2_ films with these QDs (Figure 3a,b). Thus, OAm- and MPA-passivated QDs were mainly studied in this work. In addition, as shown by the cyclic voltammetry of OAm-QD-photoanodes and MPA-QD-photoanodes (Figure S7, Supplementary Materials), the energy levels of the OAm- and MPA-passivated QDs were similar [55]. This enables a simple comparison of their PEC characteristics by mainly focusing on the adsorption mechanism of QDs to TiO_2_.

We suggest two different adsorption mechanisms for the QDs with different surface ligands (Figure 3c). For QDs passivated with monofunctional ligands (e.g., OAm-passivated QDs), the functional groups of the ligands bind to the QD surface. For these QDs, some surface ligands should be detached from the QD surface before the adsorption of QDs onto the TiO_2_. The QDs in the QD sensitized-TiO_2_ films were not washed away by the original QD solvents (e.g., dichloromethane for the OAm-passivated QDs), implying strong binding between the QDs and TiO_2_ (Figure S8, Supplementary Materials). If the surface of the QDs was fully covered by monofunctional ligands, the QDs could not be tightly bound to the TiO_2_, and they were easily removed by non-polar organic solvents. Indeed, the suggested adsorption mechanism is consistent with the results of a previous study of the adsorption mechanism of monofunctional ligand-passivated CdSe QDs on TiO_2_ for QD-sensitized solar cells [56]. The fact that QDs with strongly binding monofunctional ligands could not be efficiently adsorbed on TiO_2_ is also consistent with the proposed adsorption mechanism of monofunctional ligand-passivated QDs.

For QDs passivated with bifunctional ligands (i.e., MPA-passivated QDs in this study), one group of ligands (thiol group in this study) strongly binds to the QD surfaces, and the other group (carboxyl group in this study) can bind to the TiO_2_ surfaces [46,49,50,51]. Thus, MPA acts as a linker that bridges the QDs and TiO_2_. Owing to this adsorption mechanism, the amount of QDs adsorbed on TiO_2_ can be controlled by controlling the pH of the solution. The pH of the QD solution affects the state of the carboxyl groups. When the solution pH decreases, the proportion of ionized carboxyl groups increases. This makes MPA-passivated QDs more dispersible in polar solvents but prevents their binding to TiO_2_ surfaces. As a result, MPA-passivated QDs were more densely adsorbed on the TiO_2_ films at higher pH (Figure 3b, bottom). In a pH 14 solution, the TiO_2_–CISe QD photoanodes showed the highest adsorption density of QDs, as confirmed by their color in photographs. With this optimization, TiO_2_–CISe QD photoanodes with MPA-passivated QDs had higher QD adsorption densities than those with OAm-passivated QDs, which was verified by both the absorption spectra (Figure 3a) and photographs (Figure 3b).

**Figure 4:**
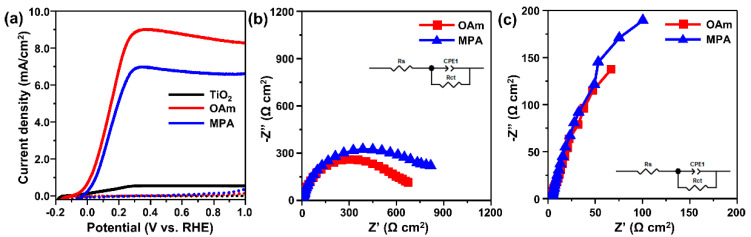
(**a**) *J*-*V* curves of an OAm-QD-photoanode, MPA-QD-photoanode, and TiO_2_ control under continuous one sun illumination (solid lines) and in the dark (dashed lines). Nyquist plots of TiO_2_–CISe QD photoanodes (**b**) in the dark and (**c**) under one sun illumination. The insets show the equivalent circuit model.

The prepared TiO_2_–CISe QD photoanodes were used for PEC hydrogen generation. The cell was composed of a conventional three-electrode system with an SCE reference electrode and a Pt rod counter electrode in an electrolyte containing 0.25 M Na_2_S and 0.35 M Na_2_SO_3_ at a controlled pH of 12.9 (Materials and Method 2.4), which is known to be an effective system for PEC hydrogen generation [39]. Anodic LSV scans were obtained to understand the PEC properties of the CISe QD-based photoanodes containing QDs with mono- and bifunctional ligands (Figure 4a). The bare TiO_2_ anodes were also measured as the control sample. The current-density–voltage curves show that all electrodes produced an anodic photocurrent from −0.2 V_RHE_ with a plateau from 0.2 V_RHE_. Both photoanodes made using MPA-passivated QDs (denoted as MPA-QD-photoanodes), and OAm-passivated QDs (denoted as OAm-QD-photoanodes) produced much higher photocurrent densities than bare TiO_2_ photoanodes. The significant enhancement in the photocurrent density demonstrates that both QDs can effectively act as additional light absorbers. The introduction of QDs greatly extended the light absorption range and intensity of TiO_2_ (Figure 3a), and the photoexcited electrons produced by QDs were transferred from the QDs to TiO_2_. For photoanodes made with OcAm-, OAc-, and DDT-passivated QDs, the photocurrents were lower than those of MPA-QD-photoanodes or OAm-QD-photoanodes (Figure S9, Supplementary Information). This was attributed to the poor sensitization of the photoanodes because of either poor colloidal stability or too strong passivation of these ligands.

The stability of the photoanodes was also tested at 0.6 V_RHE_ under light irradiation (Figure S10, Supplementary Information). About 36% of the initial photocurrent was maintained after 1 h of operation. It should be noted that increasing the stability of the QD-photoanode is usually related to the structural engineering of QDs or photoanodes rather than surface ligands, which is beyond the scope of the current study. Although the stability of the photoanodes is not as high as those using conventional CdSe QDs with structural optimization [57], we anticipate that this will be improved with future research.

Unexpectedly, the photocurrent density of an OAm-QD-photoanode (~8.2 mA/cm^2^ at 0.6 V_RHE_) is clearly higher than that of an MPA-QD-photoanode (~6.7 mA/cm^2^ at 0.6 V_RHE_) despite the high QD adsorption of the latter (Figure 4a and Table 1). Generally, the photocurrent density is approximately proportional to the adsorption density of the absorbers because more absorbers can produce more photoexcited electrons. The results suggest that electron transfer between QDs and TiO_2_ is not as effective for MPA-QD-photoanodes compared to OAm-QD-photoanodes. This is attributed to the different binding mechanisms of the QDs according to the surface ligands. The direct adsorption of Oam-QDs by partial ligand detachment was beneficial for electron transfer between QDs and TiO_2_. However, although bifunctional ligands were helpful in increasing QD adsorption, they can prevent the effective charge transfer between QDs and TiO_2_.

To gain a better understanding of the effects of surface ligands on the PEC performance, EIS analysis was performed using the TiO_2_–QD photoanodes (at 0.6 V_RHE_ in the dark state and under simulated one sun illumination). Nyquist plots (Figure 4b) were fitted using the equivalent circuit model shown in the inset, where *R*_S_ is the solution resistance, and the RC circuit represents the charge-transfer characteristics of the TiO_2_–CISe QD photoanodes and the interface between the photoanodes and electrolyte [18,58]. Consequently, *R*_ct_ is the resistance related to the charge transfer between the photoanodes and the electrolyte. As listed in Table 1, our results show that the *R*_ct_ of the MPA-QD-photoanodes was higher than that of the OAm-QD-photoanodes despite the high QD adsorption density of the MPA-QD-photoanodes. This was attributed to the presence of organic linkers between the MPA-QDs and TiO_2_, leading to the poor charge transfer between the QDs and redox couples in the electrolyte. We can expect that the resistance between the QDs and TiO_2_ is smaller in OAm-QD-photoanodes because of the direct attachment between the inorganic parts of the QDs and TiO_2_. These results imply that the better charge separation in the OAm-QD-photoanodes compared to that in the MPA-QD-photoanodes results in enhanced hole transfer from the QDs to the redox couples in the electrolyte [18,58]. In addition, the behavior of the resistance at the interface of TiO_2_–QDs is consistent with a previous spectroscopy study on electron transfer between TiO_2_ and QDs with controlled surface ligands [59]. In the literature, it was demonstrated that MPA linkers between QDs and TiO_2_ can inhibit effective electron transfer.

**Figure 5:**
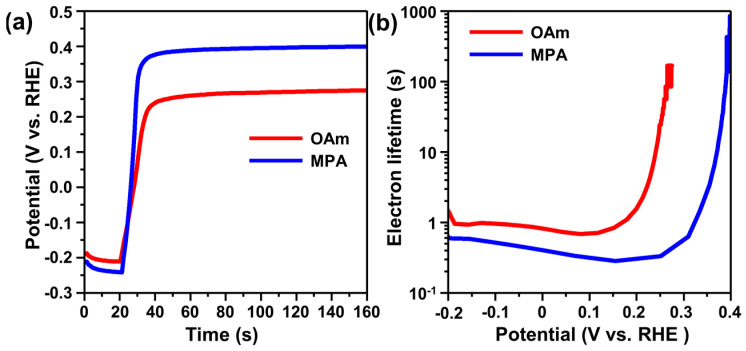
(**a**) OCVD curves and (**b**) electron lifetime as a function of V_OC_ for OAm-QD-photoanode and MPA-QD-photoanode.

We also analyzed the electron recombination kinetics in the TiO_2_–QD photoanodes with open-circuit voltage decay (OCVD) analysis, observing the decay of the open circuit voltage (V_OC_) after turning off the illumination. The OCVD curves showed decay after 20 s in the dark because of charge recombination between the charges from photoanodes and redox couples in the electrolyte (Figure 5a). The V_OC_ of the MPA-QD-photoanodes decayed more rapidly than that of OAm-QD-photoanodes. The electron lifetimes of the photoanodes were calculated from the OCVD data, and the electron lifetime versus voltage curves [60,61] are shown in Figure 5b. It is clear that the electron lifetime of the OAm-QD-photoanodes is longer (i.e., the charge recombination rate is higher) than that of MPA-QD-photoanodes. It is proposed that the organic linker molecules between the QDs and TiO_2_ in MPA-QD-photoanodes acted as defects [62], while the inorganic cores of QDs and TiO_2_ formed a direct junction in the OAm-QD-photoanodes. Considering that the major pathway of charge recombination is electron transfer from the TiO_2_ conduction band to the redox couples in the electrolyte [33,49], these defects at the interface of OAm-QDs and TiO_2_ can act as recombination centers, leading to inferior PEC performance.

The EIS and OCVD data were consistent with the proposed QD adsorption mechanism of each photoanode: (i) A direct contact was formed between the QDs and TiO_2_ in OAm-QD-photoanodes, and (ii) the QDs and TiO_2_ were connected by linker molecules in the MPA-QD-photoanodes. The direct contact between the QDs and TiO_2_ results in efficient electron transfer between them, which is also consistent with the results of a previous spectroscopy study on electron transfer between TiO_2_ and QDs with controlled surface ligands [59]. This also leads to enhanced hole transfer between the QDs and redox couples in the electrolyte and an increase in the electron lifetime in the photoanode. These results explain why the OAm-QD-photoanodes produced a high photocurrent despite the lower QD adsorption density. It should also be noted that OAm may not be the optimal surface ligand for PEC using QDs. Our findings imply that electron transfer between the QDs and TiO_2_ is important for PEC hydrogen production, which requires the careful design of the surface states of QDs.

## 4. Conclusions

This work studied the effects of the surface ligands of colloidal CISe QDs on the fabrication of PEC photoelectrodes and their resulting PEC characteristics. In particular, OAm- and MPA-passivated CISe QDs were carefully chosen for this investigation to comprehend the effects of mono- and bifunctional ligands on PEC hydrogen production employing CISe QDs. TiO_2_–QDs photoanodes were prepared by adsorbing QDs onto mesoporous TiO_2_, and the surface ligands affected the QD adsorption process. Inorganic cores of OAm-passivated QDs were directly adsorbed on TiO_2_ by partial ligand detachment, which is beneficial for electron transfer from QDs to TiO_2_. Bifunctional ligands can act as linkers by bridging QDs and TiO_2_, and the amount of QD adsorption was higher for MPA-passivated QDs than for OAm-passivated QDs. With this tradeoff, OAm-QD-photoanodes and MPA-QD-photoanodes showed current densities of ~8.2 mA/cm^2^ and ~6.7 mA/cm^2^, respectively, at 0.6 V_RHE_ under one sun illumination. These findings suggest that not only the QD adsorption density but also the electron transfer between QDs and TiO_2_ are critical for PEC hydrogen production. Our results highlight the importance of surface-ligand engineering of QDs for effective PEC hydrogen production.

## Figures and Tables

**Table 1:** Summary of *J*–*V* characteristics and impedance analysis for TiO_2_—QDs photoanodes.

Sample	Current Density (mA/cm^2^)	Dark R_s_ (Ω cm^2^)	Dark R_ct_ (Ω cm_2_)	Light R_s_ (Ω cm^2^)	Light R_ct_ (Ω cm_2_)
OAm-QD-photoanode	8.236	4.25	660.8	3.21	1041
MPA-QD-photoanode	6.740	3.37	857.2	3.23	1180

All data were measured at 0.6 V_RHE_.

## Data Availability

All data are contained within the article.

## Supplementary Materials

The following supporting information can be downloaded at: https://www.mdpi.com/article/10.3390/ma15176010/s1, Figure S1: Tauc Plot. Figure S2: TEM images of CISe QDs. Figure S3: XRD patterns of CISe QDs. Figure S4: Absorption spectra and photographs of QD solutions with various surface ligands. Figure S5: Cross-sectional SEM analysis of OAm-QD-photoanodes. Figure S6: Cross-sectional SEM analysis of MPA-QD-photoanodes. Figure S7: Cyclic voltammograms. Figure S8: Washing tests. Figure S9: Additional *J-V* curves of QD-photoanodes. Figure S10: *J-t* plot.
