# Supplementary material for: Effects of Mono- and Bifunctional Surface Ligands of Cu–In–Se Quantum Dots on Photoelectrochemical Hydrogen Production
Source: Materials (Basel). 2022 Aug 31;15(17):6010. doi: 10.3390/ma15176010 (PMC9457290; doi:10.3390/ma15176010)
Supplement: Supplementary file 1 [file materials-15-06010-s001.zip › materials-1842237-supplementary.pdf]

materials

MDPI

Supplementary Materials

Effects of Mono- and Bifunctional Surface Ligands of Cu-In-Se Quantum Dots on Photoelectrochemical Hydrogen Production

Soo Ik Park [1,†], Sung-Mok Jung [2,†], Jae-Yup Kim [2,*] and Jiwoong Yang [1,3,*]

1 Department of Energy Science and Engineering, Daegu Gyeongbuk Institute of Science and Technology (DGIST), Daegu 42988, Republic of Korea
2 Department of Chemical Engineering, Dankook University, Yongin 16890, Republic of Korea
3 Energy Science and Engineering Research Center, Daegu Gyeongbuk Institute of Science and Technology (DGIST), Daegu 42988, Republic of Korea
* Correspondence: jykim@dankook.ac.kr (J.-Y.K.); jiwoongyang@dgist.ac.kr (J.Y.)
† These authors contributed equally to this work.

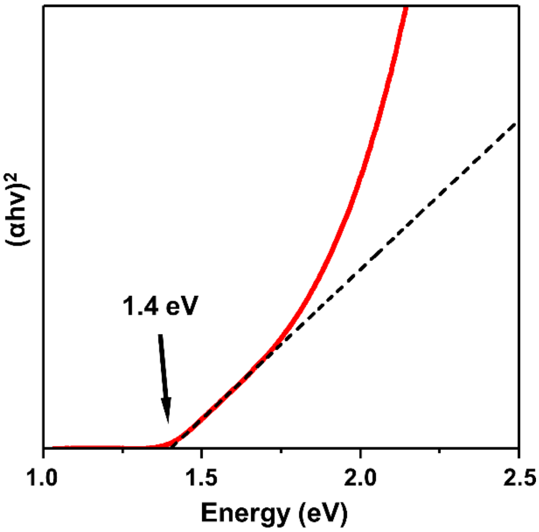

Figure S1. Tauc plot of CISe QDs. The plot was acquired from the absorption spectrum of CISe QDs to determine the bandgap energy.

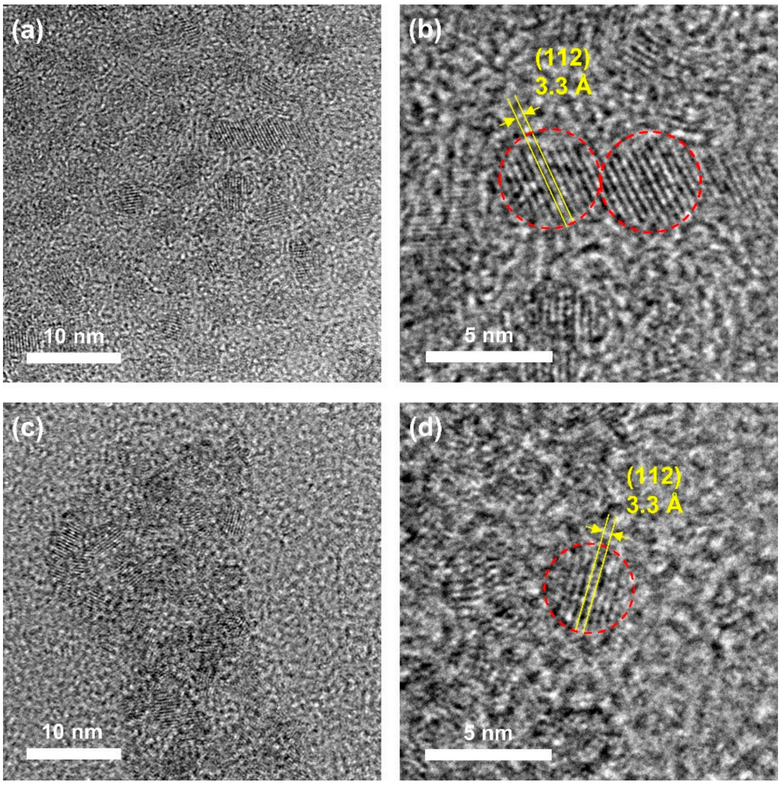

Figure S2. TEM analysis of CISe QDs. (**a-b**) OAm-passivated CISe QDs, and (**c-d**) MPA-passivated CISe QDs. The OAm- and MPA-passivated CISe QDs present the same size, shape, and crystal structure.

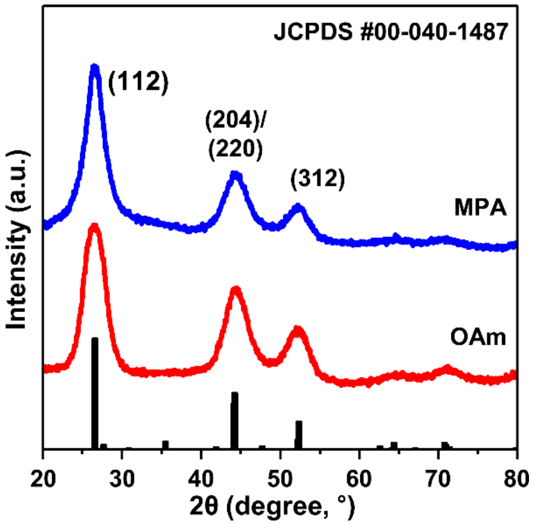

Figure S3. The XRD patterns of OAm- and MPA-passivated CISe QDs.

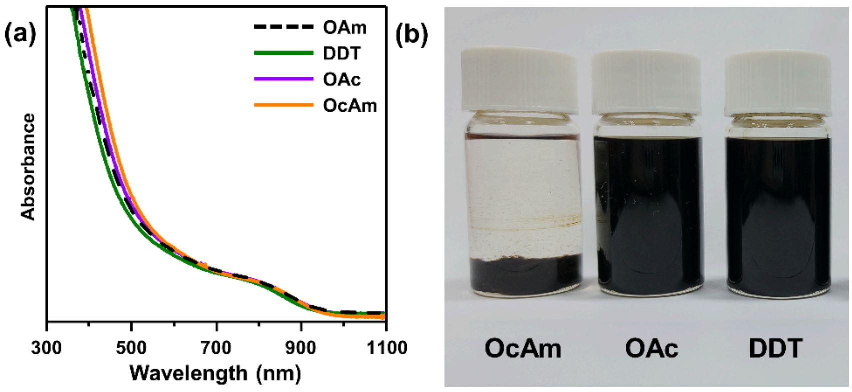

Figure S4. (**a**) Absorption spectra and (**b**) photographs showing the colloidal solution of OcAm-, DDT-, and OAc-passivated CISe QDs in dichloromethane.

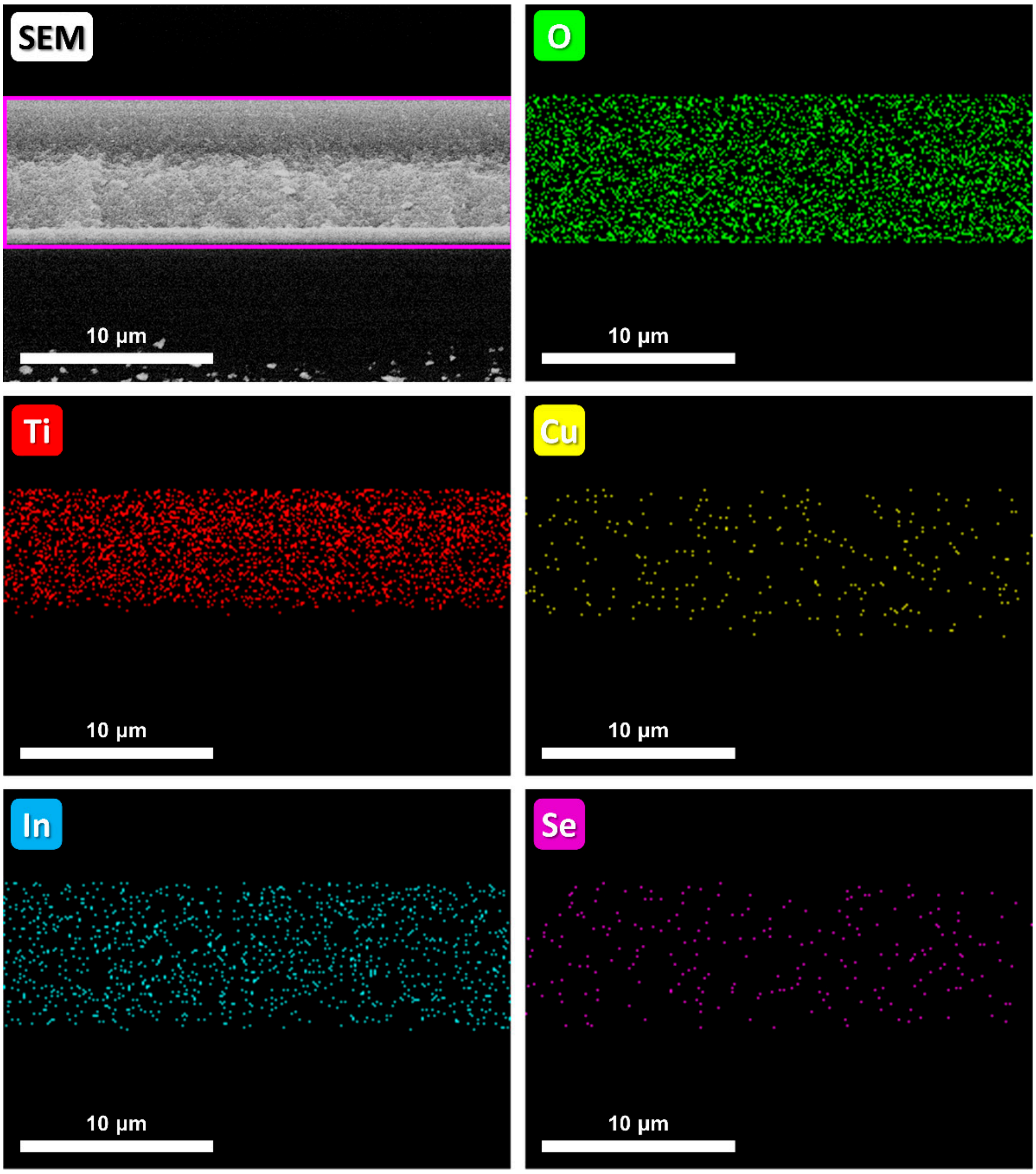

Figure S5. Cross-sectional SEM image and the corresponding energy-dispersive spectroscopy (EDS) elemental mapping of an OAm-QD-photoanode.

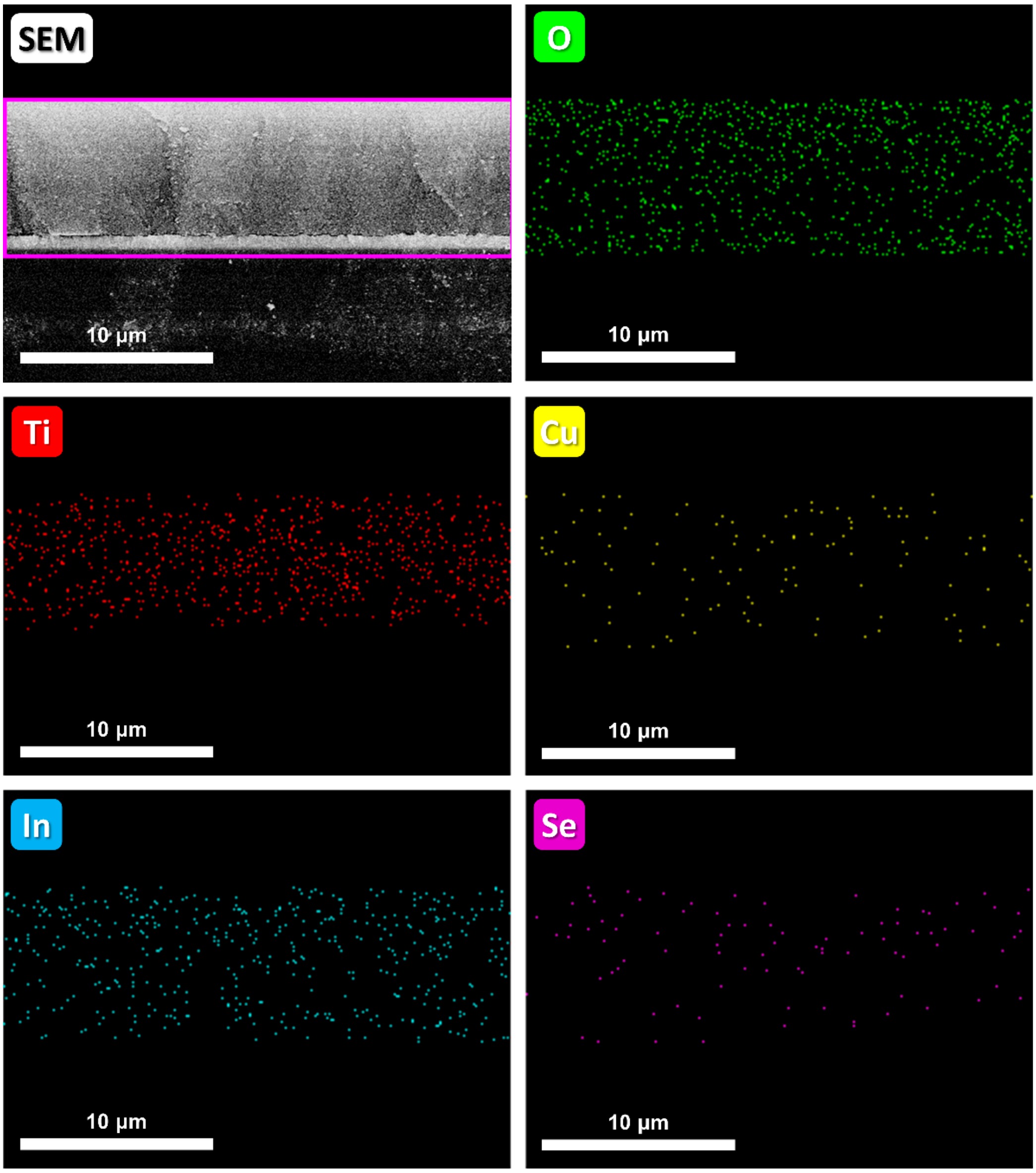

Figure S6. Cross-sectional SEM image and the corresponding EDS elemental mapping of an MPA-QD-photoanode.

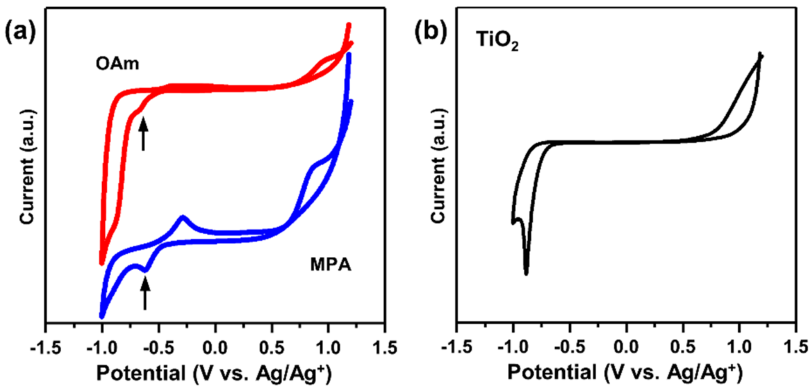

Figure S7. (**a**) Cyclic voltammograms of OAm-QD-photoanodes and MPA-QD-photoanodes. The arrows indicate the first reduction peaks of CISe QDs, which is related to their conduction band minimum. (**b**) Cyclic voltammogram of bare TiO_2 photoanodes.

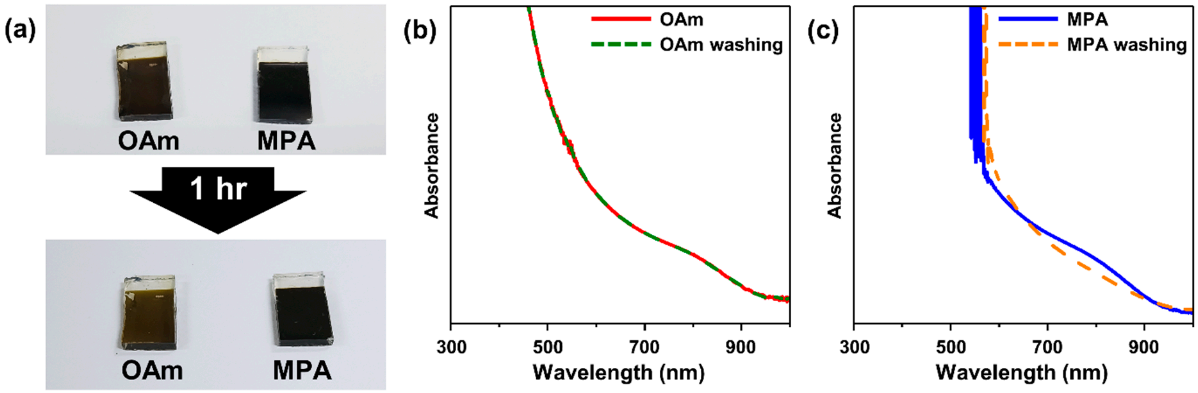

Figure S8. (**a**) Photographs and (**b**,**c**) absorption spectra comparing QD sensitized-TiO_2 films before and after the washing (each film was dipped into the original solvent for 1 h).

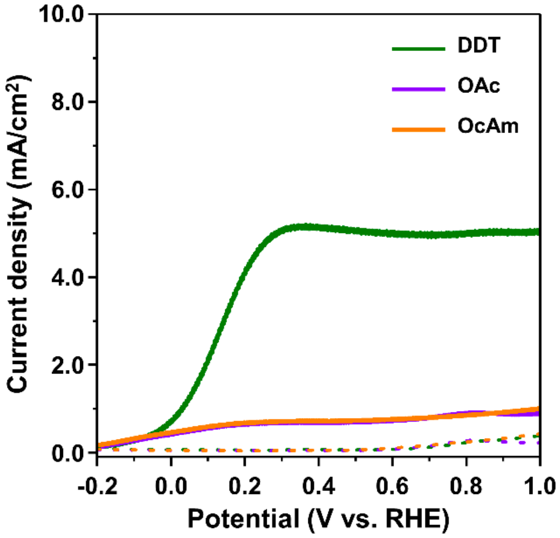

Figure S9. *J-V* curves of QD-photoanodes made by OcAm-, DDT-, and OAc-passivated CISe QDs. The data were collected under continuous one sun illumination.

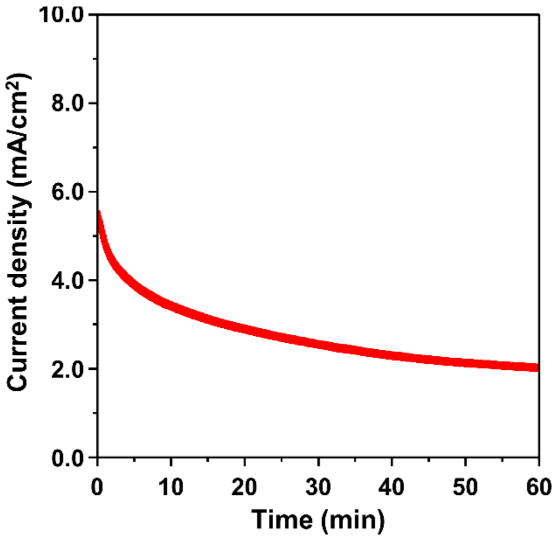

Figure S10. *J*-*t* plot (at 0.6 V_{RHE}) of the OAm-QD-photoanodes.
